# Acute stress differentially affects spatial configuration learning in high and low cortisol-responding healthy adults

**DOI:** 10.3402/ejpt.v4i0.19854

**Published:** 2013-05-02

**Authors:** Thomas Meyer, Tom Smeets, Timo Giesbrecht, Conny W. E. M. Quaedflieg, Harald Merckelbach

**Affiliations:** Faculty of Psychology and Neuroscience, Maastricht University, Maastricht, The Netherlands

**Keywords:** Maastricht Acute Stress Test, contextual cueing paradigm, spatial memory, hippocampal area, posttraumatic stress disorder

## Abstract

**Background:**

Stress and stress hormones modulate memory formation in various ways that are relevant to our understanding of stress-related psychopathology, such as posttraumatic stress disorder (PTSD). Particular relevance is attributed to efficient memory formation sustained by the hippocampus and parahippocampus. This process is thought to reduce the occurrence of intrusions and flashbacks following trauma, but may be negatively affected by acute stress. Moreover, recent evidence suggests that the efficiency of visuo-spatial processing and learning based on the hippocampal area is related to PTSD symptoms.

**Objective:**

The current study investigated the effect of acute stress on spatial configuration learning using a spatial contextual cueing task (SCCT) known to heavily rely on structures in the parahippocampus.

**Method:**

Acute stress was induced by subjecting participants (N = 34) to the Maastricht Acute Stress Test (MAST). Following a counterbalanced within-subject approach, the effects of stress and the ensuing hormonal (i.e., cortisol) activity on subsequent SCCT performance were compared to SCCT performance following a no-stress control condition.

**Results:**

Acute stress did not impact SCCT learning overall, but opposing effects emerged for high versus low cortisol responders to the MAST. Learning scores following stress were reduced in low cortisol responders, while high cortisol-responding participants showed improved learning.

**Conclusions:**

The effects of stress on spatial configuration learning were moderated by the magnitude of endogenous cortisol secretion. These findings suggest a possible mechanism by which cortisol responses serve an adaptive function during stress and trauma, and this may prove to be a promising route for future research in this area.

Acute stress and stress-related hormonal activity profoundly influence learning and memory. Such stress-induced memory alterations have attracted the attention of researchers, many of whom believe that they typically serve adaptive purposes but can also represent key mechanisms in the development of highly prevalent emotional disorders (de Kloet, Joels, & Holsboer, 2005; de Quervain, Aerni, Schelling, & Roozendaal, 2009; Joels, 2011; Wingenfeld & Wolf, 2011). Indeed, different mood and anxiety disorders appear to be characterized by abnormal memory function, such as enhanced learning, consolidation, or retrieval of negative information (de Quervain et al., 2009; Wolf, 2008), as well as more distressing involuntary recollections (Brewin, Gregory, Lipton, & Burgess, 2010). In posttraumatic stress disorder (PTSD), stress-related changes in memory function are of particular relevance, as this disorder may develop after highly stressful events and is characterized by recurrent flashbacks and an apparent inability to integrate these aversive memories with other autobiographical memories (Brewin et al., 2010).

Based on studies investigating stress effects on learning in rodents (e.g., Schwabe, Schachinger, de Kloet, & Oitzl, 2010) and humans (e.g., Schwabe et al., 2007), Schwabe and colleagues (Schwabe, Wolf, & Oitzl, 2010) recently suggested that stress may reduce “cognitive” memory formation in the hippocampal area (e.g., spatial navigation learning) in favor of increased reliance on “habit” memory (e.g., associations based on stimulus–response). Interestingly, these findings accord well with a recent theory on the development of intrusions (Brewin et al., 2010) proposing that intrusions of stressful experiences occur when a memory system in the hippocampal area fails to construct contextualized representations of that event (i.e., possibly as a consequence of stress). Therefore, investigating the effects of stress on memory formation in the hippocampal area may provide further theoretical insights with relevance for stress and trauma research.

In humans, this line of research has focused almost exclusively on (declarative) memory of words or pictures (for review, see Schwabe, Wolf, et al., 2010), whereas little attention has been given to stress effects on visuo-spatial memory until very recently (Taverniers et al., 2011; Taverniers, Taylor, & Smeets, in press). Nevertheless, some studies indicate that visuo-spatial learning, based on the hippocampal area, is relevant to PTSD symptoms. For instance, PTSD patients displayed impaired spatial configuration processing, a deficiency that was statistically predictive of PTSD symptom severity (Gilbertson et al., 2007). Another study using trauma films in healthy individuals found that view-point independent spatial recognition performance predicted fewer intrusions (Bisby, King, Brewin, Burgess, & Curran, 2010). In light of theories suggesting reduced hippocampal-area based memory under stress, and a role of this brain region in intrusions (c.f., supra), these findings imply that stress and stress hormone responding could be related to intrusions by impairing visuo-spatial learning efficiency.

In accordance with the Gilbertson et al. (2007) and Bisby et al. (2010) studies, a recent study by Meyer et al. (in press) demonstrated that intrusions after viewing a trauma film were related to worse implicit spatial configuration learning in a contextual cueing paradigm (Chun & Jian, 1998). This paradigm measures the degree to which spatial configurations of multiple simple cues are bound in memory, which depends crucially on structures in the medial temporal lobe (Chun & Phelps, 1999; Manns & Squire, 2001). An fMRI study in healthy participants (Preston & Gabrieli, 2008) has indicated that this implicit learning performance relies on structures in the parahippocampus, which are thought to serve as a major input for the construction of spatial representations in the hippocampus (Fyhn, Hafting, Treves, Moser, & Moser, 2007). Although the exact relationship between contextual cueing performance and intrusions remains to be clarified, visuo-spatial learning appears to reflect information processing that is adaptive during a traumatic event. The current study addresses whether this performance is affected by stress and stress hormone responses.

To test this, we compared performance on a contextual cueing paradigm under stress and no-stress control conditions. Specifically, healthy individuals were subjected to the Maastricht Acute Stress Test (MAST; Smeets et al., 2012) and to a no-stress version of the MAST in a counterbalanced within-subject cross-over design, measuring subjective and hormonal (i.e., cortisol) responses on each test occasion. Both conditions were followed by the administration of a contextual cueing task, allowing us to assess the effects of stress versus control condition within subjects.

To our knowledge, this is the first human study to address stress effects on implicit visuo-spatial learning known to rely on parahippocampal structures. In contrast, prior studies focused on word or picture learning (often finding memory facilitation when stress targets the learning phase; for a review, see Schwabe, Wolf, et al., 2010) or on explicit spatial memory performance (finding memory impairments when stress targets the learning phase; Taverniers et al., 2011, in press). Given the scarcity of prior research, it was not possible a priori to formulate firm hypotheses. However, based on theoretical considerations (c.f., supra), we expected contextual cueing performance to be worse in the stress condition as a result of heightened glucocorticoid responses. Since the literature suggests possible gender differences in the effect of stress on memory (e.g., Smeets, Dziobek, & Wolf, 2009; Wolf, Schommer, Hellhammer, McEwen, & Kirschbaum, 2001), we tested this in a balanced sample of men and women.

## Method

### Participants

A total of 34 healthy participants (50% women), recruited at Maastricht University campus, completed this study. Mean age was 21.4 (*SD*=3.4, range = 18–36). Eligibility was checked using a screening form, exclusion criteria being a body mass index (BMI; kg/m^2^) below 18 or above 30, cardiovascular disease, severe physical illness, endocrine disorders, current psychopathology, substance abuse, heavy smoking (>10 cigarettes/day), and current use of medication known to affect the function of the hypothalamic–pituitary–adrenal (HPA) axis. For women, hormonal contraceptive use was required as an inclusion criterion, as this is known to suppress cortisol response variation due to the menstrual cycle (Kudielka, Hellhammer, & Wüst, 2009). The standing ethical committee of the Faculty of Psychology and Neuroscience, Maastricht University, approved this study. All participants gave written informed consent and were compensated with a small financial reward or partial course credit in return for their participation.

### Maastricht Acute Stress Test

The MAST (Smeets et al., 2012) is an effective stress induction procedure that combines physical stress with uncontrollability, unpredictability, social–evaluative, and mental arithmetic elements to produce reliable subjective and cortisol stress responses. The duration of the MAST is 15 min. Participants first undergo a 5-min preparation phase in which instructions about the MAST procedure are presented on a computer screen. In the following 10-min acute stress phase, they are alternately prompted by instructions on the computer screen to immerse their hand in ice-cold water (2°C) or to engage in a mental arithmetic test (counting backwards from 2043 in steps of 17). During mental arithmetic trials, participants are additionally asked to direct their gaze toward a video camera (enabling them to see themselves on a TV monitor) and receive negative performance feedback by the experimenter concerning accuracy and/or speed of the calculations. In the current study, five hand immersion trials (duration: 60 or 90 sec) were alternated with four mental arithmetic trials (duration: 45–90 sec), while participants were unaware of the number and exact duration of the two types of trials.

### No-stress control condition

The procedure of the no-stress control condition was identical to the MAST, except that all stressful elements were removed (see Smeets et al., 2012; Experiment 3). That is, there was no videotaping and the water was lukewarm (35°C). The mental arithmetic test was replaced by instructions to count aloud consecutively from 1 to 25 at a self-chosen pace and to start again at 1 when having reached 25. The experimenter checked participants’ compliance but provided no feedback on their performance.

### Assessment of stress responses

**Fig. 1:**
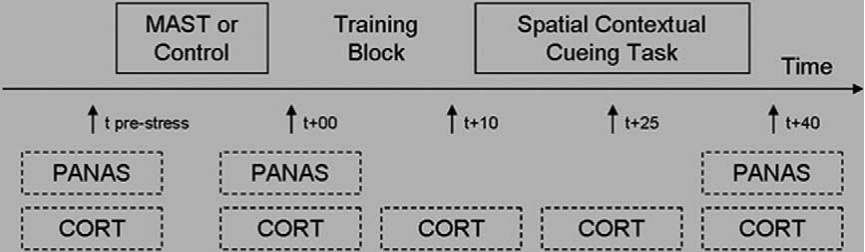
Overview of the procedure. The procedure was similar for session 1 and session 2, the crucial difference being the stress vs. control condition. MAST, Maastricht Acute Stress Test; PANAS, Positive and Negative Affect Schedule; CORT, cortisol sample.

Mood changes in response to the MAST and control condition were measured using repeated administrations of the Positive and Negative Affect Schedule, state version (PANAS; Watson, Clark, & Tellegen, 1988), consisting of two 10-item subscales for positive affect (PA; all Cronbach's alpha > 0.88) and negative affect (NA; all Cronbach's alpha > 0.69). Items refer to current mood (e.g., PA: *interested*, NA: *distressed*) and are rated on five-point scales (1 = *very slightly or not at all*; 5 = *very much*). Salivary cortisol measurements were taken as a measure of hormonal stress responding of the HPA axis using synthetic Salivette devices (Sarstedt^®^, Etten-Leur, the Netherlands) at five time points in each session. The first measurement was taken immediately before MAST or control condition onset (i.e., 15 min before stress offset; *t*_pre-stress_) and four times relative to the end of the MAST or control condition (*t*_+00_, *t*_+10_, *t*_+25_, and *t*_+40_; see Fig. 1). On collection, samples were stored at -20°C immediately. Cortisol levels were determined by a commercially available luminescence immuno assay (IBL, Hamburg, Germany). Mean intra- and inter-assay coefficients of variation are typically less than 8 and 12%, respectively, and the lower and upper detection limits were 0.015 mg/dl (0.41 nmol/l) and 4.0 mg/dl (110.4 nmol/l), respectively.

### Spatial Contextual Cueing Task

In the contextual cueing paradigm (Chun & Jian, 1998), participants are required to find a single target (“T”-shaped symbol rotated 90° or 270°) among 11 rotated “L”-shaped distracters (i.e., the visuo-spatial context of the target). In half of the trials, the configuration of distracter stimuli is repeated, while in the other half of the trials new distracter configurations are presented. The repeated target contexts predict the location of the target, thereby facilitating search, as evidenced by faster reaction times (RTs) compared to new distracter configurations. This RT difference is a measure of the contextual learning effect (Chun & Jian, 1998), with higher values reflecting a stronger learning effect.

Similar to Meyer et al. (in press), we used the abbreviated Spatial Contextual Cueing Task (SCCT) developed by Bennett and colleagues (2009). Each SCCT administration consisted of 30 blocks with 12 trials, 6 of which had repeated arrays, while 6 had novel arrays. In addition, 12 trials with new arrays were presented along with task instructions as a separate training block before the actual task. All arrays were computed individually for each participant and session. Trials started with a 1 sec fixation period, followed by a configuration display that required participants to indicate as quickly and accurately as possible whether the base of the target stimulus pointed left or right by pressing response keys on a right-hand response box. The configuration display was presented for 10 sec or until the participant responded. Before the next trial started, auditory feedback was provided upon response (a high-pitch or low-pitch tone for correct or incorrect/too slow responses, respectively). Each block was followed by a break that could be ended by the participant. After block 15, there was a forced 2-min break for the collection of a saliva cortisol probe.

For data reduction, median RTs of accurate trials were derived for each of the 30 blocks, and separately for each array type (novel and repeated). These median RTs were subsequently averaged across five consecutive blocks, respectively, yielding novel and repeated RT scores of six epochs. For each of the six epochs, one learning score was then calculated by subtracting repeated RT scores from novel RT scores. In addition, accuracy scores were calculated for the six epochs, and separately for array type.

### Procedure

Participants were invited to two lab sessions separated by a 1-week interval. Each session took place in the afternoon between 12:30 and 18:00 h to control for circadian cortisol rhythms (Nicolson, 2008). Before each session, participants were instructed by email to come well-rested, to refrain from consuming alcohol or drugs the evening before participation, and to refrain from other activities known to affect cortisol measurements immediately before participation (e.g., eating, smoking, heavy physical activity, brushing teeth). Adherence to these rules was checked upon arrival, and in cases of violation the session was rescheduled (this applied to only one participant). Next, participants were subjected to either the MAST or the control condition, preceded also by administration of the PANAS and a salivary cortisol probe. Participants were then given SCCT instructions and one SCCT training block. Participants performed the SCCT, with cortisol probes being taken before, midway through, and after task administration. Finally, the PANAS was administered a third time. The procedure of the second session was similar to the first one, except that MAST and a control condition were substituted (see Fig. 1; with counterbalanced order of MAST and control condition across participants).

### Statistical analysis

Affective (i.e., PA and NA) and cortisol responding was analyzed using repeated measures ANOVAs with time point of measurement and condition (MAST, control) as within-subjects factors. To explore gender effects, sex was additionally entered as between-subjects factor, and where appropriate, follow-up tests and post-hoc pairwise comparisons with Bonferroni adjustment were used to explore interaction effects. In addition, delta-peak cortisol values from the stress condition were used to compare the magnitude of cortisol responding between males and females, using an independent-samples *t*-test. The effect of condition on SCCT learning scores was analyzed using repeated measures ANOVA with epoch and condition as within-subjects factors and gender as between-subjects factor. To assess whether possible reductions in contextual cueing performance can be accounted for by glucocorticoid responses in the stress condition, cortisol responding was entered by creating groups of low and high cortisol responders based on delta-peak cortisol values relative to pre-stress (group allocation by median-split). Also, we tested a linear association between delta-peak cortisol values and mean SCCT learning scores of the stress condition using correlational analysis. SCCT accuracy scores were not included in the analyses because they were too close to ceiling in all arrays, epochs, and conditions (all means > 97%). When the assumption of non-sphericity was violated in the data, Greenhouse–Geisser corrected degrees of freedom and *p*-values are reported. Alpha was set at 0.05 for all tests.

## Results

### Cortisol responses

**Fig. 2:**
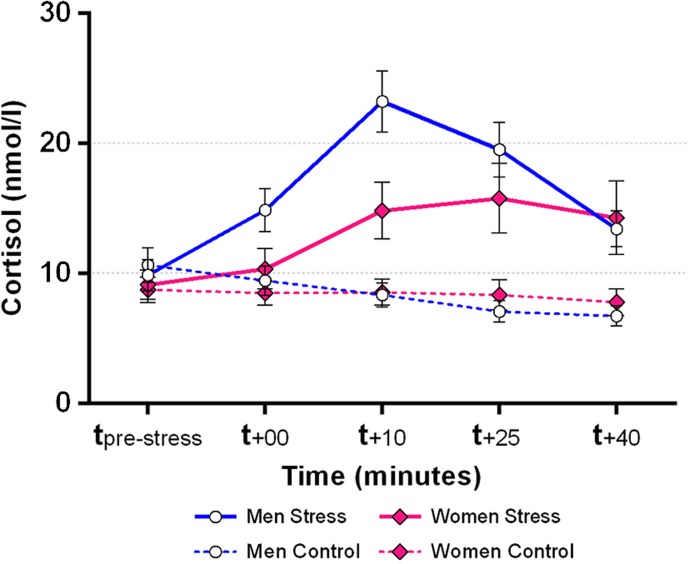
Cortisol responses in men and women to the MAST vs. control condition. Endogenous cortisol responses were robust in both men and women using hormonal contraceptives; error bars represent standard errors of measurement.

A 2 (Condition: stress, control) by 5 (Time: cortisol measurements) by 2 (Gender) repeated measures ANOVA revealed a significant three-way interaction of Time by Condition by Gender, *F*(1.9, 59.9) = 4.7, *p*=0.014, $\eta_{p}^{2}$=0.13. Separate follow-up tests for men and women revealed significant Time by Condition interactions in both men, *F*(2.1, 33.3) = 23.8, *p*<0.001, $\eta_{p}^{2}$=0.60 and women, *F*(1.5, 24.2) = 6.2, *p*=0.011, $\eta_{p}^{2}$=0.28. Post-hoc pairwise comparisons showed that men displayed elevated cortisol levels in the stress condition, as compared with the control condition, at *t*_+10_, *t*_+25_, and *t*_+40_ (Bonferroni-adjusted *p*s < 0.005), but not at *t*_pre-stress_ or *t*_+00_ (adjusted *p*s > 0.115). Relative to the control condition, women displayed elevated cortisol levels in the stress condition only at *t*_+10_ and *t*_+25_ (adjusted *p*s < 0.036), but not at *t*_pre-stress_, *t*_+00_, or *t*_+40_ (adjusted *p*s > 0.118). An independent-samples *t*-test comparing delta-peak cortisol levels in the stress condition between men and women revealed a trend toward stronger cortisol increases in men (*M*=14.5 nmol/l, *SD*=9.5) than in women (*M*=8.5 nmol/l, *SD*=9.2), *t*(32) = 1.9, *p*=0.069. Descriptively, 88% of men (15/17) and 65% of women (11/17) could be classified as cortisol responders in the MAST condition (i.e., displaying a cortisol increase ≥ 2.5 nmol/l; e.g., Kirschbaum, Pirke, & Hellhammer, 1993), Pearson Chi-square = 2.62, *p*=0.106. Cortisol data are summarized visually in Fig. 2.

### Mood responses

A 2 (Condition: stress, control) by 3 (Time: PANAS measurements) by 2 (Gender) repeated measures ANOVA for NA revealed a significant Time by Condition interaction, *F*(1.4, 45.4) = 14.2, *p*<0.001, $\eta_{p}^{2}$=0.31, in the absence of main or interactive effects involving Gender (all *F*s < 2.3, all *p*s > 0.143). Post-hoc pairwise comparisons indicated that NA levels were elevated in the stress condition, as compared with the control condition, at pre-stress, *t*(33) = 2.6, Bonferroni-adjusted *p*=0.042, and at post-stress, *t*(33) = 4.6, adjusted *p*<0.001. NA levels did not differ between conditions at the end of the session, *t*(33) = 0.9, *ns*. For PA, the 2 by 3 by 2 repeated measures ANOVA revealed a main effect of Time, *F*(2, 64) = 24.2, *p*<0.001, $\eta_{p}^{2}$=0.43, that did not interact with Condition, Gender, or both (all *F*s < 1.6, all *p*s > 0.22). Post-hoc pairwise comparisons showed that in both conditions, PA decreased from pre- to post-stress, *t*s(33) > 3.0, Bonferroni adjusted *p*s < 0.002, and remained stable afterwards, *t*(33) < 2.2, Bonferroni adjusted *p*s > 0.127.

### Stress effects on SCCT performance^1^

A 2 (Condition: stress, control) by 6 (Epoch) by 2 (Gender: male, female) repeated measures ANOVA on SCCT learning scores revealed a significant main effect of Epoch, *F*(3.2, 103.5) = 10.1, *p*<0.001, $\eta_{p}^{2}$=0.24, with contextual learning scores increasing across epochs. This learning effect did not interact with Condition, *F*(4, 129.3) = 0.73, *p*=0.58, $\eta_{p}^{2}$=0.02, Gender, *F*(3.2, 103.5) = 2.3, *p*=0.08, $\eta_{p}^{2}$=0.07, or both, *F*(4, 129.3) = 1.1, *p*=0.38, $\eta_{p}^{2}$=0.03. There were also no main effects of Condition, *p*=0.90, or Gender, *F*(1, 32) = 1.5, *p*=0.22, $\eta_{p}^{2}$=0.05. Overall, average SCCT learning scores differed significantly from zero (*Grand Mean*=181.9 ms; *SE*=42.0), *F*(1, 32) = 18.7, *p*<0.001, $\eta_{p}^{2}$=0.37, reflecting the typical contextual cueing effect.

**Fig. 3:**
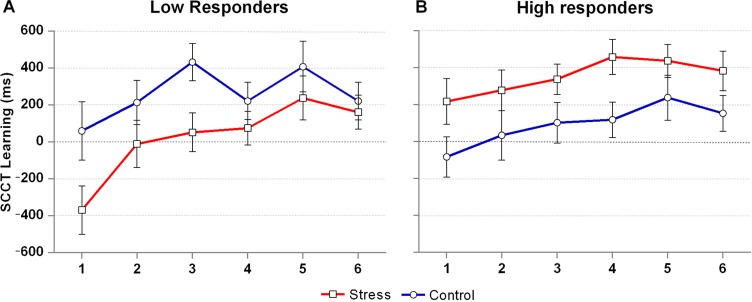
Contextual cueing effect across time for low and high cortisol responders. *N*=17 in both groups. SCCT Learning scores reflect the RT difference on trials with novel and repeated arrays, respectively, with higher values indicating better learning. Error bars represent standard errors of measurement.

To assess the specific role of cortisol responding, delta-peak cortisol values were entered as a two-level factor (group allocation by median-split).^2^ A 2 (Condition: stress, control) by 6 (Epoch) by 2 (Responder: high, low) repeated measures ANOVA showed a significant Condition by Responder interaction, *F*(1, 32) = 9.2, *p*=0.005, $\eta_{p}^{2}$=0.22, in the absence of a three-way interaction, *F*(3.9, 126) = 0.9, *p*=0.46. Examination of this effect separately for each condition showed that the responder groups differed from each other in the stress, *F*(1, 32) = 8.1, *p*=0.008, $\eta_{p}^{2}$=0.203, but not the control condition, *F*(1, 32) = 1.9, *p*=0.177, $\eta_{p}^{2}$=0.06. Follow-up tests suggest a *negative* effect of the stress condition as compared with the control condition on SCCT learning in *low* cortisol responders, *F*(1, 16) = 4.9, uncorrected *p*=0.042, $\eta_{p}^{2}$=0.23, and a *positive* effect of the stress condition in *high* cortisol responders, *F*(1, 16) = 4.4, uncorrected *p*=0.052, $\eta_{p}^{2}$=0.22 (see Fig. 3). In line with these findings, delta-peak cortisol values in the stress condition correlated positively with SCCT learning scores in the stress condition, *r*=0.353, *p*=0.042 (two-tailed).

## Discussion

The present study explored the effects of stress and stress-related cortisol secretion on implicit spatial configuration learning in humans. We used a contextual cueing paradigm that requires a type of learning that is thought to depend crucially on the parahippocampal region (Chun & Phelps, 1999; Manns & Squire, 2001; Preston & Gabrieli, 2008). In a counterbalanced within-subject cross-over design, participants were subjected to the MAST (Smeets et al., 2012) and to a no-stress control condition, each condition followed by administration of the SCCT (Bennett et al., 2009). Based on the proposition that hippocampal-area based memory may be reduced under stress (Schwabe, Wolf, et al., 2010), we hypothesized that stress would undermine implicit spatial configuration learning.

Demonstrating the effectiveness of our stress task, we observed both subjective (in terms of NA increases from pre- to post-stress) and hormonal (in terms of salivary cortisol increases) stress responses in the MAST condition, but not in the control condition. In both sessions, participants displayed the contextual cueing effect (i.e., faster RTs on trials with repeated vs. novel arrays), as well as an increase of this effect in the course of the task, which is typical for this paradigm (Bennett et al., 2009; Chun & Jian, 1998). Our data also suggest that SCCT administration itself did not affect stress responding, since the SCCT was neither accompanied by cortisol increases nor by negative mood responses in the control condition.

Contrary to our expectations, there was no overall effect of the stress condition on the contextual cueing effect or on learning across epochs, compared to the control condition. There also was no evidence suggesting that the effect of stress would differ between men and women, which adds to a literature with mixed results with respect to gender differences (e.g., Smeets et al., 2009; Wolf et al., 2001). When taking cortisol secretion into account, however, opposing effects of the stress condition emerged for high versus low responders, which turned out to account for the absence of an overall condition effect in the sample. In particular, our data suggest that only participants with low cortisol secretion (including non-responders) have reduced overall SCCT learning scores after stress, whereas in participants with higher cortisol secretion, our data suggest a trend towards amplified learning scores after stress. In support of this interpretation, we found a significant positive correlation between cortisol responding and mean SCCT learning scores in the stress condition.

Importantly, in both low and high cortisol responders, stress appeared to have main effects on SCCT learning scores (i.e., diminishing or amplifying the overall contextual cueing effect), whereas the increase of learning scores over time was unaffected by stress in both responder groups. This indicates that stress had an effect on an early stage of implicit spatial memory formation, which was apparently moderated by endogenous cortisol secretion, with continued visuo-spatial learning in the course of the task apparently remaining unaffected. Although studies using the contextual cueing paradigm typically do not distinguish between stages of implicit spatial memory formation (e.g., Chun & Jian, 1998), these findings seem to suggest that initial encoding of spatial configurations can be modulated independently from further consolidation of these acquired memories. It has been argued that stress can affect consolidation (in long-term declarative memory) through influencing the degree of rehearsal (e.g., Tollenaar, Elzinga, Spinhoven, & Everaerd, 2008). Because the contextual cueing paradigm inherently controls the number of occasions at which repeated spatial configurations are rehearsed (i.e., each repeated array is presented exactly 30 times), this could be the reason why no stress effects on further consolidation of the contextual cueing effect were found.

Our findings do not support the hypothesis that acute stress impairs implicit spatial configuration learning in general, but point to a specific moderating role of endogenous cortisol secretion. Notably, with respect to explicit memory, differential effects of stress, depending on cortisol responding, have been reported (see, e.g., Domes, Heinrichs, Reichwald, & Hautzinger, 2002; Smeets et al., 2009). These cortisol-dependent opposite effects of stress on implicit visuo-spatial learning may have implications for theories of stress and stress-related psychopathology. For instance, the prediction of reduced reliance on “cognitive” (including declarative spatial) learning in the hippocampal area as a consequence of cortisol increases (Schwabe, Wolf, et al., 2010) apparently does not translate to a reduced contextual cueing effect under stress. Instead, we found that cortisol secretion protected or even amplified learning. This might indicate that cortisol differentially affects different systems in the hippocampal area that subserve spatial memory formation. In particular, previous human studies have largely addressed stress effects on declarative memory for which the hippocampus is crucial (Schwabe, Wolf, et al., 2010), whereas the contextual cueing critically depends on structures in the parahippocampus (Chun & Phelps, 1999; Manns & Squire, 2001), notably the entorhinal cortex (Preston & Gabrieli, 2008). These regions serve as major input to the hippocampus for the construction of spatial representations (Fyhn et al., 2007) and may thus display differential responses to stress. However, it is also possible that even more extreme levels of cortisol secretion (e.g., in response to strenuous Special Forces exercises; Taverniers et al., in press) would lead to lowered performance in the contextual cueing paradigm, as hormonal stress effects have often been hypothesized to follow an inverted-U quadratic function (e.g., Abercrombie, Kalin, Thurow, Rosenkranz, & Davidson, 2003).

Interestingly, several studies have reported reduced resting (i.e., basal) cortisol concentrations in PTSD patients, and a link between lowered cortisol and the development and maintenance of intrusions has been suggested (for a review, see Wingenfeld & Wolf, 2011). In line with this, administration of low doses of cortisol has been shown to be a promising treatment option in PTSD patients (e.g., de Quervain & Margraf, 2008). Since cortisol elevations during delayed retrieval typically impair declarative memory performance (Schwabe, Wolf, et al., 2010), hypocortisolism in PTSD has been argued to result in weaker inhibition of trauma memories and, hence, to cause more intrusions (Wingenfeld & Wolf, 2011; though note that lowered resting cortisol does not necessarily imply smaller cortisol stress responses). Our results, however, revealed that strongly enhanced cortisol secretion during stress dampens or even reverses the negative effects of stress on spatial configuration learning. This might reflect a different mechanism by which cortisol responding has adaptive consequences under stress. In addition to inhibiting the retrieval of trauma memories, cortisol might enhance adaptive information processing associated with spatial configuration learning. Relevant to this, Meyer et al. (in press) recently found that superior performance on the SCCT was negatively correlated with intrusions in healthy participants who had viewed a trauma film. Although it is not yet clear in what way implicit visuo-spatial learning would help to reduce intrusions, it might thus reflect information processing that is relevant to the formation of contextualized trauma memories. In this way, stronger cortisol responses during stressful experiences might help to integrate the trauma in autobiographical memory and prevent intrusions, which could be a promising avenue for future research.

## Limitations

The current study had some limitations that deserve to be mentioned. To begin with, our sample consisted entirely of young healthy adults, and it is not clear whether our findings would apply to other populations. Also, although the MAST is a relatively robust experimental stressor (Smeets et al., 2012), the effects may not be comparable to real-life traumatic stressors, implying that these findings may not directly translate to PTSD patients per se. Another limitation is that the design of this study allows no conclusions about differential effects of stress on encoding and consolidation of spatial configurations on the one hand, and on retrieval on the other hand. That is, the repetition of configurations in the SCCT can, by definition, invoke all three of these processes simultaneously. However, it might be possible in future studies to disentangle the effects of stress on encoding and retrieval by delivering a delayed test that focuses on a long-term component of implicit spatial memory (e.g., Chun & Jiang, 2003). Finally, although our findings suggest a specific moderating role of endogenous cortisol secretion in the effects of stress on spatial configuration learning, we are not able to infer whether this role is causal or merely correlational. Therefore, future studies are required to test the possible causal involvement of cortisol by experimentally manipulating hormonal responding (e.g., using pharmacological interventions).

## Conclusions

A large body of evidence shows that stress and stress hormones affect hippocampal-area based memory in various ways that are relevant to our understanding of stress-related psychopathology, including PTSD (de Kloet et al., 2005; Joels, 2011; Wingenfeld & Wolf, 2011). Recent evidence shows that human spatial processing and learning is also affected by stress (Taverniers et al., 2011, in press) and might be involved in PTSD symptoms (Bisby et al., 2010; Gilbertson et al., 2007; Meyer et al., in press). The current study demonstrates that stress affects implicit visuo-spatial learning relying on structures in the parahippocampus, whereby the level of endogenous cortisol secretion appeared to moderate the effect of stress on learning performance. The memory-enhancing role of higher cortisol levels on this memory system during stress may indicate that stress has different effects on hippocampal and parahippocampal components of spatial memory. These findings suggest a possible mechanism by which cortisol responses serve as an adaptive function during stress and trauma, which may inspire future studies in this area.

## Footnotes

1 The procedural order of Condition (MAST first vs. control condition first) had no significant main or interaction effects on SCCT learning and was therefore not included in subsequent analyses.

2 Using area under the curve with respect to increase (AUC_I_; Pruessner, Kirschbaum, Meinlschmid, & Hellhammer, 2003) for Responder group allocation yielded nearly identical groups and results.
